# PLA with Intumescent System Containing Lignin and Ammonium Polyphosphate for Flame Retardant Textile

**DOI:** 10.3390/polym8090331

**Published:** 2016-09-05

**Authors:** Aurélie Cayla, François Rault, Stéphane Giraud, Fabien Salaün, Vanessa Fierro, Alain Celzard

**Affiliations:** 1 Univ. Lille Nord de France, F-59000 Lille, France; francois.rault@ensait.fr (F.R.); stephane.giraud@ensait.fr (S.G.); fabien.salaun@ensait.fr (F.S.); 2 ENSAIT, GEMTEX, F-59100 Roubaix, France; 3 Institut Jean Lamour–UMR CNRS-Université de Lorraine n 7198, ENSTIB, F-88026 Epinal cedex, France; vanessa.fierro@univ-lorraine.fr (V.F.); alain.celzard@univ-lorraine.fr (A.C.)

**Keywords:** PLA, lignin, ammonium polyphosphate, flame retardant, melt spinning

## Abstract

Using bio-based polymers to replace of polymers from petrochemicals in the manufacture of textile fibers is a possible way to improve sustainable development for the textile industry. Polylactic acid (PLA) is one of the available bio-based polymers. One way to improve the fire behavior of this bio-based polymer is to add an intumescent formulation mainly composed of acid and carbon sources. In order to optimize the amount of bio-based product in the final material composition, lignin from wood waste was selected as the carbon source. Different formulations of and/or ammonium polyphosphate (AP) were prepared by melt extrusion and then hot-pressed into sheets. The thermal properties (thermogravimetric analyses (TGA) and differential scanning calorimetry (DSC)) and fire properties (UL-94) were measured. The spinnability of the various composites was evaluated. The mechanical properties and physical aspect (microscopy) of PLA multifilaments with lignin (LK) were checked. A PLA multifilament with up to 10 wt % of intumescent formulation was processed, and the fire behavior of PLA fabrics with lignin/AP formulation was studied by cone calorimeter.

## 1. Introduction

In recent years, the development, functionalization and use of biopolymers have become of growing interest. Indeed, the traditional petroleum-based plastics generate environmental concerns related in particular to their end of life (pollution) and raise some questions about their future availability in regard to limited and depleted natural oil resources. Therefore, the worldwide use of bioplastics is expected to register an annual growth rate of 41.4% [1], resulting from a consumer demand for more eco-friendly polymers. Among all biopolymers, polylactic acid (PLA) is one of the most promising candidates. This thermoplastic aliphatic polyester derived from sustainable resources such as corn, potato, cane molasses, and sugar beets [2] has received more and more attention because it can be used as a substitute for petroleum-based commodity polymers such as polystyrene, polyester, etc. in a wide range of applications. Thus, PLA can be used in various application fields such as packaging, automotive, textile and so on, and be processed in different forms including extruded films, injection-molded products, fibers and fabrics.

According to the required application in the textile field, staple fibers, short-cut fibers, and continuous mono- or multifilaments of PLA can be used to produce knitted or woven fabrics and wet-laid, spunbond or meltblown nonwovens [3]. PLA filaments and fiber-reinforced composites were first used in medical applications (absorbable sutures, artificial implants, etc.) as a result of their degradability and biocompatibility properties. Then, in order to substitute polyethylene terephtalate (PET), the use of PLA was extended to the clothing industry (sportswear, and ballistic-protective clothing), to furnishing (upholstery, and carpets) or to composite materials. Even if it is reported that PET and PLA yarns have relatively similar properties, some differences can be noticed—in particular concerning fire retardancy behavior: the limit of the oxygen index of PLA fibers (24–30) is higher than PET (20–21) [4], and an improvement in self-extinguishing behavior is observed for PLA fibers, accompanied by a decrease in smoke generation (63 m^3^ kg^−1^ vs. 394 m^3^ kg^−1^) and fewer combustion calories (4500 kcal kg^−1^ vs. 5500 kcal kg^−1^). Together, these factors result in a lower flammability in PLA compared to PET [5,6]. Nevertheless, PLA still presents certain flame retardant properties that are insufficient for some applications with more stringent requirements.

Throughout the past decade, a number of studies have been performed to improve the fire behavior of PLA through functionalization. Most of them have been carried out on films, plates or composites with various retardant additives such as phosphorous compounds, halogenated compounds, nitrogenated compounds, inorganic compounds, materials containing silicon or carbon (expanded graphite), etc. [7,8]. Flame retardant PLA textiles can be developed following three ways: (i) by using nonreactive systems [9,10]; (ii) by using reactive systems; and (iii) by developing inherently flame retardant fiber. Thus, Solarski et al. [11,12] have introduced various clay nanoparticles (Cloisite^®^ 30B from Southern Clay or Bentone 104 from Elementis Specialties) into PLA by melt blending in order to obtain knitted fabric from multifilament yarns. The cone calorimeter experiments carried out on these textile structures have shown out an increase in char yields as well as a lower peak in the heat release rate. Over the last decade, the development of fire retardant biopolymers has received great attention due to the desire to promote a “green concept”. Thus for, the formulation of intumescent flame retardant systems (IFR) may be based on bio-based acidic sources (fumaric acid [13], phytic acid [14,15]) or bio-based carbonization agents (chitosan [14,16], sorbitol [17], xylitol [17], cyclodextrin [17,18], starch [17,19,20,21,22,23]). Among those entirely bio-based carbonization agents, lignin has also been considered by some researchers [19,20,24,25,26,27] due to its abundance among polymers in nature. Thus, Reti et al. [19,20] and Zhang et al. [24,25,26] have prepared composites containing between 20 and 40 wt % of IFR composed of lignin and ammonium polyphosphate (AP) to enhance the flame retardancy of PLA.

To our knowledge, there are no studies in the literature dedicated to intumescent flame retardant PLA fibers. The aim of this present work is to study different formulations of PLA with lignin and ammonium polyphosphate using a conventional melt compounding process, in contrast to previous evaluations of the spinnability of PLA filled with lignin and/or ammonium polyphosphate to manufacture knitted structures. The thermal properties and fire behavior of composites were established by thermogravimetric analyses and vertical burning tests (UL-94), and the flame retardancy of knitted structures was investigated by cone calorimeter experiments.

## 2. Materials and Methods

### 2.1. Materials

The PLA grade (NatureWorks^®^ 6202D) was chosen for its spinnability by melt processes and was purchased from Cargill–Dow (Midland, MI, United States). The lignin used is a kraft lignin (LK) supplied by Institut Jean Lamour (laboratory linked with ENSTIB, Ecole Nationale Supérieure des Technologies et Industries du Bois, Epinal, France). This lignin was kindly supplied by Innventia (Stockholm, Sweden) and is presently commercialized under the name LignoBoost by the company Metso. LK is precipitated from softwood black liquor by the injection of CO_2_. It is next filtered, re-dispersed, acidified again, filtered once more and finally washed. As a result, a much purer lignin than usual is obtained, having very low carbohydrate, sulphur and ash contents, typically 0.5–1.5, 1–3, and 0.2–1 wt % respectively on a dry basis [28]. Additional details can be found elsewhere [29]. Ammonium polyphosphate (AP) (Exolit AP 422) purchased from Clariant (Choisy-le-Roi, France) was used as the acidic source in the intumescent system and is a fine white powder with an average particle size of 15 μm and an onset decomposition temperature of 275 °C.

### 2.2. Processing

#### 2.2.1. Extrusion

Samples of different PLA/LK/AP ratios were prepared using a ThermoHaake (Thermo Fisher Scientic, Waltham, MA, USA) co-rotating, intermeshing twin-screw extruder (*L*/*D* = 25) according to Table 1. The mixtures were dried at 60 °C for 24 h before extrusion. The screws′ rotational speed was fixed at 100 rpm for all blends. The extruder included five heating zones in which the temperature was independent and fixed on a graduated scale from 170 to 190 °C. The extruded blends were air-granulated to be transformed (sheets, multifilaments) or characterized (MFI, TGA, DSC).

#### 2.2.2. Melt Spinning

Pellets obtained through the extrusion process were fed into the hopper of the melt spinning machine (SPINBOY I driver, Busschaert Engineering, Deerlijk, Belgium), before drying for 12 h at 60 °C. After passing through the single-screw extruder, they were injected through holes with a diameter of 1.2 mm by a volumetric pump rotating at 16 rpm to ensure a constant flow. Two bundles of 40 monofilaments were obtained which were then cooled in air and combined into a multifilament. The final melt-spun multifilament thus consisted of 80 monofilaments and which was coated in spin finish oil and rolled up on two heated rolls at varying speeds (S1 and S2) to ensure a draw. The theoretical draw is given by the draw ratio DR = S2/S1. The spinning parameters of the melt spinning device are shown in Table 2 where T1 to T7 corresponds to the temperatures from the hopper to the die.

#### 2.2.3. Knitting

Due to their structural properties (thickness, drape, density, etc.) and easy processability, knitted textile structures were preferred to woven and nonwoven fabrics. The Milano structure [30] was chosen for all the formulations to perform cone calorimeter characterization. All the knitted fabrics were equally thick at 2 mm and had a grammage of 1300 ± 50 g m^−2^.

### 2.3. Characterizations

#### 2.3.1. Melt Flow Index

The Melt Flow Tester from ThermoHaake was used for MFI measurement to analyze spinnability and determine the spinning temperature condition. According to the standard ASTM D1238 [31], the piston and the dried material (7 g per measurement) were pre-heated for 4 and 3 minutes, respectively. This procedure was carried out twice for each blend at different temperatures (190 and 220 °C) depending on the blends tested under a load of 2.16 kg.

#### 2.3.2. Thermal Analyses

Thermogravimetric (TG) analyses were carried out on TA 2050 Instruments (TA Instruments, New Castle, DE USA). The samples (weight of 10 ± 0.5 mg) were heated under nitrogen (flow rate: 50 mL min^−1^) at a controlled speed (10 °C min^−1^ up to 600 °C). The onset thermal degradation temperature (Td “onset”) which is measured for 5 wt % loss due to degradation was calculated, as well as also the temperature at the maximal degradation point (Tdmax) and the residue content (R in %) at 500 °C. The accuracy of the measurement device was estimated at ±0.3% of weight loss, which is slightly higher than the accuracy of the balance given by TA Instruments (±0.1%). The TG curve and its derivative (DTG) were plotted for each sample. The thermal characteristics of the blends were investigated and curves of weight difference between the experimental and theoretical TG curves were computed following Equations (1) and (2):

(1) $$\Delta (M(T))=M_{\exp}(T)-M_{theo}(T)$$

(2) $$M_{theo}(T)=(Wt{\%}_{PLA}\times M_{\exp}{\left(T\right)}_{PLA}+Wt{\%}_{LK}\times M_{\exp}{\left(T\right)}_{LK}+Wt{\%}_{AP}\times M_{\exp}{\left(T\right)}_{AP})/100$$

where Δ(*M*(*T*)) is the curve of residual mass difference; *M*_exp_(*T*) is the experimental residual mass of blends (variation by temperature T); *M_theo_*(*T*) is the theoretical residual mass of the blends; *Wt*%*_PLA_* is the weight percentage of virgin PLA; *Wt*%*_LK_* is the weight percentage of LK; *Wt*%*_AP_* is the weight percentage of AP; *M*_exp_(*T*)*_PLA_* is the experimental residual mass of virgin PLA; *M*_exp_(*T*)*_LK_* is the experimental residual mass of LK; and *M*_exp_(*T*)*_AP_* is the experimental residual mass of AP.

The Δ(*M*(*T*)) curves allow the observation of any increase or decrease in the thermal stability of the formulations compared to the combination of components analyzed separately.

Differential scanning calorimetry (DSC) characterizations of each multifilament were performed on a 2920 Modulated DSC (TA Instruments, New Castle, DE, USA) with typically 10 ± 0.1 mg of dry material. The manipulation carried out under nitrogen atmosphere (with a flow of 50 mL min^−1^) consisted of two identical cycles, the first being devoted to the elimination of the thermal history of the composite. Each cycle run as follows: from 20 to 230 °C at 10 °C min^−1^, an isotherm of 5 min at 200 °C and the cooling scan at 10 °C min^−1^ to return to 20 °C. Analyses were made on the second cycle. Melting enthalpies (Δ*H_m_*) and temperatures (Tm), cold crystallization enthalpies (Δ*H_CC_*) and cold temperatures (Tcc), and glass temperatures (Tg) were determined from the heating scan. The crystallinity degree (χ) of the PLA was calculated according to Equation (3):

(3) $$\chi (\%)=\frac{\Delta H_{m}-\Delta H_{cc}}{\Delta H_{m}^{0}(1-x)}$$

where $\Delta H_{m}^{0}$ is the reference enthalpy defined as the heat of a 100% crystalline sample. The thermodynamic enthalpy of crystallization used for crystallinity calculation is 93.6 J g^−1^ [32] and x is the content of LK and/or AP.

#### 2.3.3. Microscopy

A cross section of multifilament yarn was observed and a digital camera coupled with a binocular microscope (Axiolab Pol from Carl Zeiss, Jena, Germany) captured pictures of the cuttings. Three points of the outline of every filament are marked, and the software proceeds to an interpolation to obtain the diameter. In each, 10 monofilament diameters are determined to obtain a representative average value of every multifilament.

Scanning electron microscopy (SEM) was performed by UMET laboratory from Université Lille I (France), to obtain information on the morphology of blends. The surfaces were carbon-coated prior to observation and analyzed using a SEM Hitachi S4700 (Schaumburg, IL, USA) operating at 6 kV and 15 mA.

#### 2.3.4. Mechanical Properties

The tensile properties of monofilaments extracted from the multifilament yarn were determined out following standard NF EN ISO 5079 [33] on a Zwick tensile testing machine (1456) (Ulm, Germany); the cell force used was 10 N. All of the tests were made at standard atmosphere (the temperature was 20 ± 2 °C and the relative humidity 65% ± 5%). The length of the sample was 20 mm and the deformation rate 20 mm min^−1^. The results given (strain and stress at break, and tenacity) represent an average value of ten tests.

#### 2.3.5. Fire Testing

Evaluations of the flammability properties of different sheets (100 × 13 × 3 mm^3^) were performed by UL-94. The samples were hung vertically, with the bottom of the sample placed 30 cm above a container with cotton. A flame was applied to the lower ending of the sample for ten seconds. After the ten seconds, the flame was removed and the burning time was measured. If the sample was not completely burned after the extinction of the flame, the process was repeated. The samples were weighed before and after the test in order to obtain the percentage of mass lost (weight loss in percent calculated according to Equation (4)). Each composition was tested three times.

Percentage of mass lost = 100 × Residual mass/Initial mass
(4)

Cone calorimetry was employed to investigate the combustion behaviors of knitted fabrics (100 × 100 × 2 mm^3^) at an incident radiant flux of 25 kW m^−2^, following standard NF ISO 5660 [34]. The experiments were done at the CREPIM (Centre de REcherche Pour l′Ignifugation des Matériaux, Bruay-la-Buissière, France). Three experiments were held for each formulation and the results presented are the averages. Data on the principal fire properties were obtained: time of ignition (t_ign_), peak of heat release rate (PHRR), total heat released (THR), total smoke volume (TSV) and residual mass. From HRR values, we calculated the average rate of heat emission (ARHE), defined as Equation (5), and its maximum (MARHE).

(5) $$ARHE(t_{n})=\frac{\sum_{2}^{n}(t_{n}-t_{n-1})\times \frac{q_{n}+q_{n-1}}{2}}{t_{n}-t_{0}}$$

where t_n_ is the time (s), t_0_= 0 s; and q_n_ is the rate of heat released at t_n_ (MJ m^−2^).

## 3. Results and Discussion

### 3.1. Melt Spinnability of Binary System: PLA-LK

**Figure 1:**
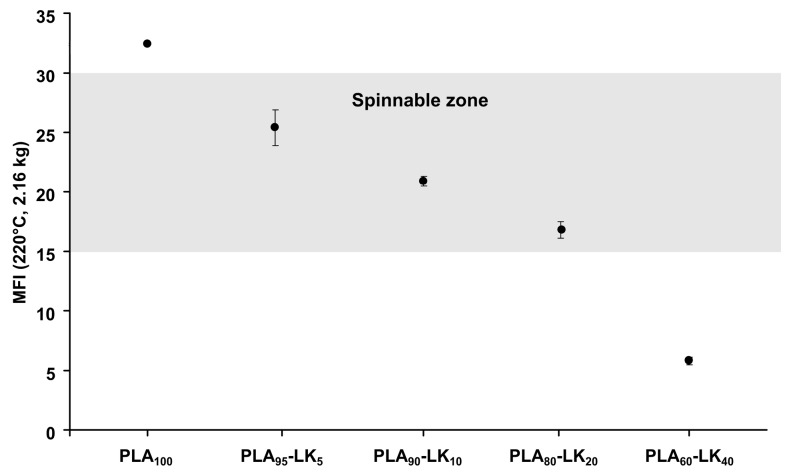
Melt flow index (MFI), (220 °C, 2.16 kg) of PLA and PLA-LK pellets.

The spinnability of the various PLA/LK blends was evaluated from the melt flow index (MFI). Figure 1 shows MFI values according to the lignin content in the PLA. The MFI test was realized at 220 °C, meaning above the melt spinning temperature of PLA (190 °C), to fluidize the blends. A progressive decrease in MFI values with increased LK content indicates a modification of the rheological properties of PLA. Extensive cross-linking and strong intramolecular interactions of polymeric lignin constrain the utilization of polymeric lignin in solid material systems, but these interactions can be disrupted in polymer blending, thus altering the viscoelastic properties of lignin. However, observations of miscible polymer blends with lignin are few detailed in the literature [35,36]. This leads to a weak development of fiber based on lignin by melt spinning process. Thunga et al., have worked on the esterification of lignin to improve the processability of lignin blends thanks to an enhanced miscibility of lignin and PLA in blends [37]. In the present study, the lignin selected was not miscible with PLA. The same trend regarding increased viscosity has been observed with introduction of lignin in low-density polyethylene (LDPE), linear low-density polyethylene (LLDPE), high-density polyethylene (HDPE) and atactic polystyrene (PS) [38]. According to the MFI results, spinnability was achieved for compositions up to 20 wt % LK in PLA to obtain multifilaments.

**Figure 2:**
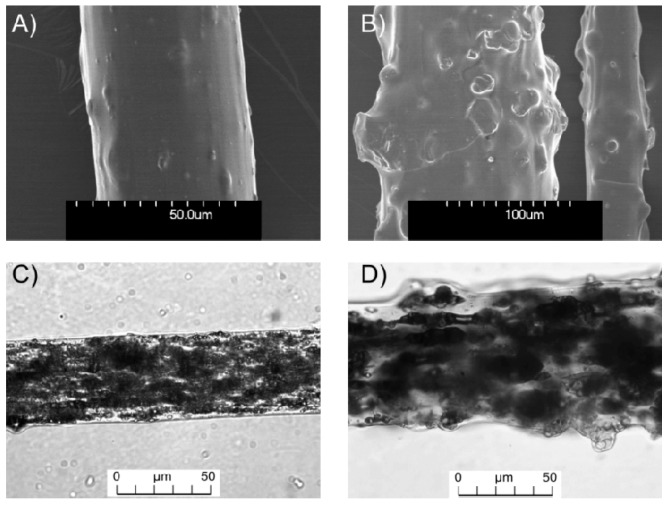
Scanning electron microscopy (**A**,**B**) and optical microscope (**C**,**D**) observations of PLA_95_-LK_05_ (**A**,**C**) and PLA_80_-LK_20_ (**B**,**D**) multifilaments.

The morphology, physical properties, and surface state of the multifilaments are strongly influenced by the loading content of lignin (Table 3 and Figure 2). Thus, incorporation of LK leads not only to the formation of irregularities on multifilaments, as can be seen on SEM and optical pictures (Figure 2), but also to an increase in the mean diameter with a high standard deviation. The high viscosity of the polymeric blends coupled with a low draw ratio (1.6) during the spinning stage lead to a poor dispersion of LK particles in the fibers, which tend to aggregate themselves and form bumps. The phenomenon is even more pronounced when the LK content increases. The fibers have an irregular rough surface, because observable particles in the range of 10–30 μm are embedded.

The mechanical properties of pure PLA filaments are a result of the semi-crystalline nature of PLA. The inclusion of lignin has an effect on the glass temperature of the multifilament samples, since it is slightly lower than that of the neat PLA sample; however, no significant differences were determined for the extrudated samples (Table 4). This may be attributed to a plasticizing effect induced by the PLA chain reorganization during the melt-spinning process. Besides, the formation of aggregated particles in the polymeric matrix creates some free volume and thus allows an increase in the chain′s mobility. PLA/LK samples are mainly amorphous under the process conditions, since little or no crystallinity not any nucleating effect is observed. The introduction of lignin affects the interactions among the PLA chains, this change greatly affects the cold crystallization phenomenon: the PLA chains have lower interaction and crystallize with greater difficulty and in a higher and broader temperature range. The LK also affects the melting of the blends, where the melting peaks are shifted to lower temperatures.

The mechanical properties of the blends are strongly influenced by the presence and the loading content of LK (Table 3). Thus, strain at break and tenacity from 10 wt % of LK are four times lower than those of neat PLA. The measured values of the sample labeled PLA_80_-LK_20_ are close to those of the PLA_90_-LK_10_ sample. The presence of particle aggregates and the poor dispersion linked to the increase of loading content are the main causes of the starting point of cracks (Figure 2). The dispersion of lignin is mainly influenced by the chemical structure of the polymeric matrix. Thus, in their study, Sallem-Idrissi et al., have selected PA-6 and succeeded in a good dispersion overall with a partial miscibility of the lignin in the matrix, allowing the mechanical properties to be maintained [36]. Adequate lignin dispersion in the polymeric matrix may lead to enhance mechanical properties such as an increased Young′s modulus due to the reduction of lignin aggregates. The introduction of lignin improves the elongation at break value up to 10 wt % due to the PLA macromolecular chain organization around lignin particles. The presence of hydroxyl groups in the lignin allows the creation of hydrogen bonds with the PLA matrix, which induces more entanglement as the content of lignin increases. Nevertheless, from 10 wt %, the decrease of elongation at break value suggests that the presence of larger agglomerates induces lower mechanical properties, undoubtedly due to the creation of free volume of initial crack. Even if the mechanical properties of composite filaments seem to be weak, they are sufficient to allow knitting.

### 3.2. Thermal and Fire Characterizations of PLA-LK-AP Composites and Fabrics

**Figure 3:**
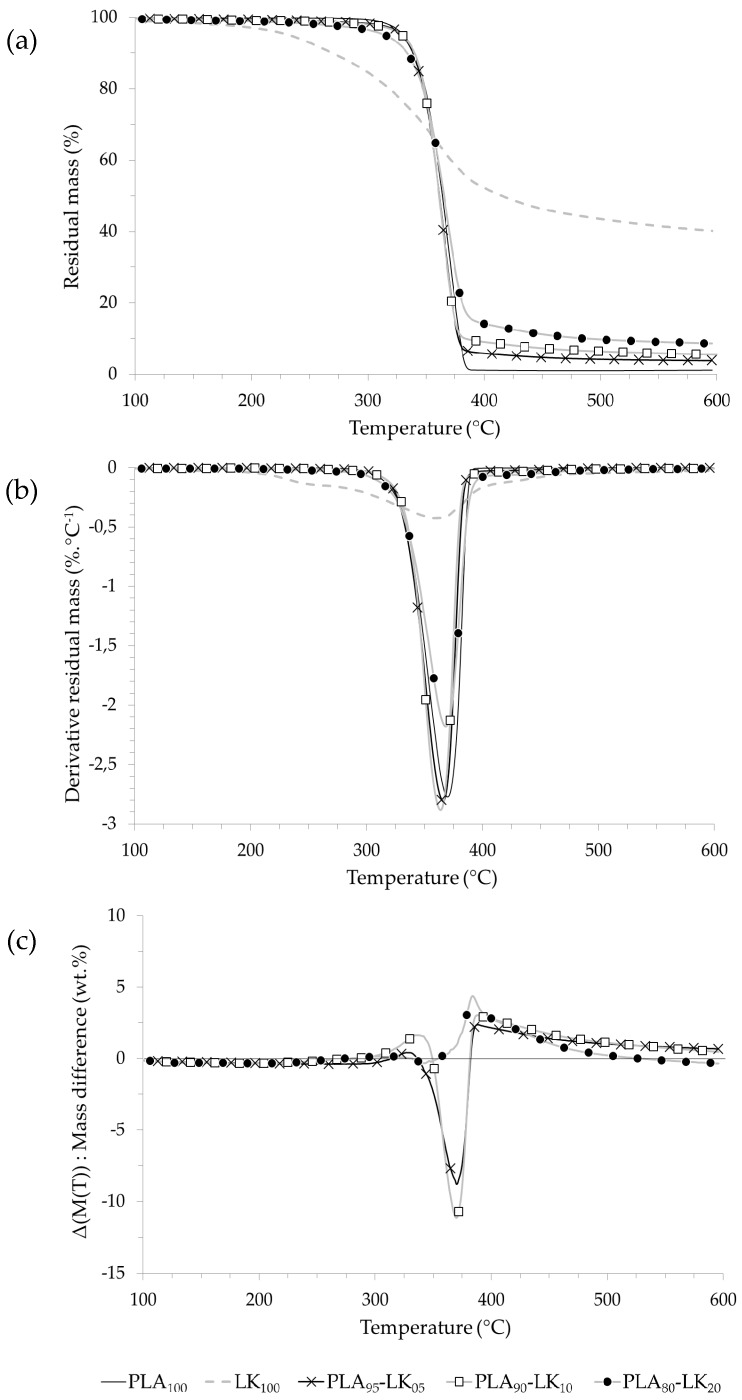
(**a**) Thermogravimetric (TG); (**b**) derivative thermogravimetric (DTG); and (**c**) mass difference curves of PLA-LK composites.

**Figure 4:**
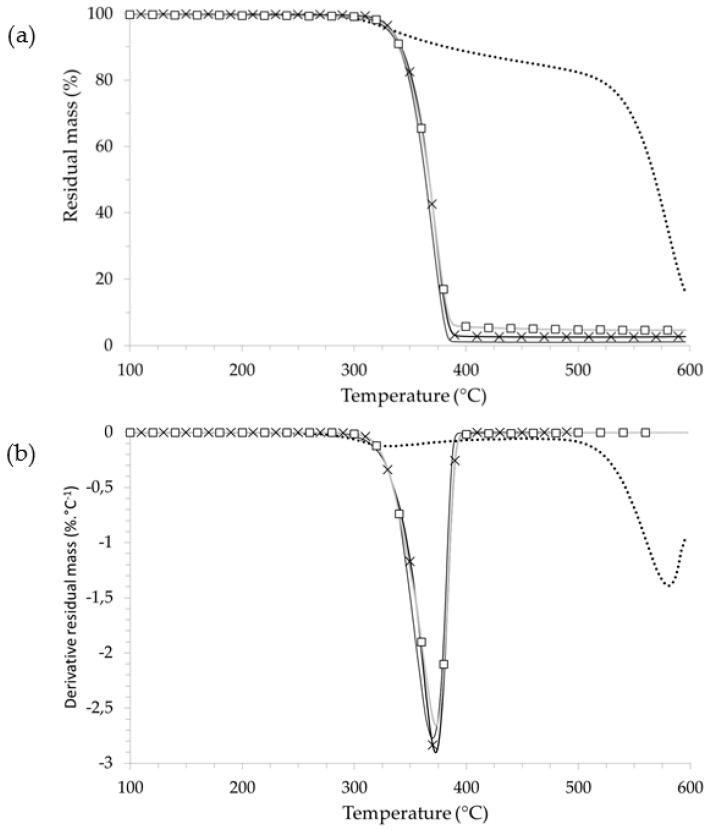

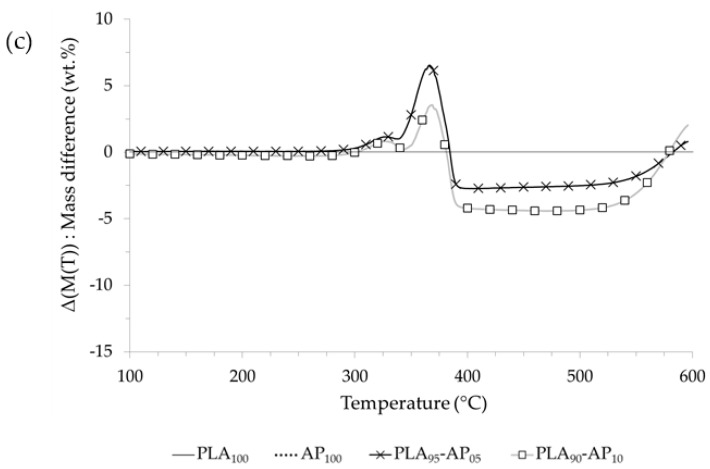
(**a**) TG; (**b**) DTG; and (**c**) mass difference curves of PLA-AP composites.

**Figure 5:**
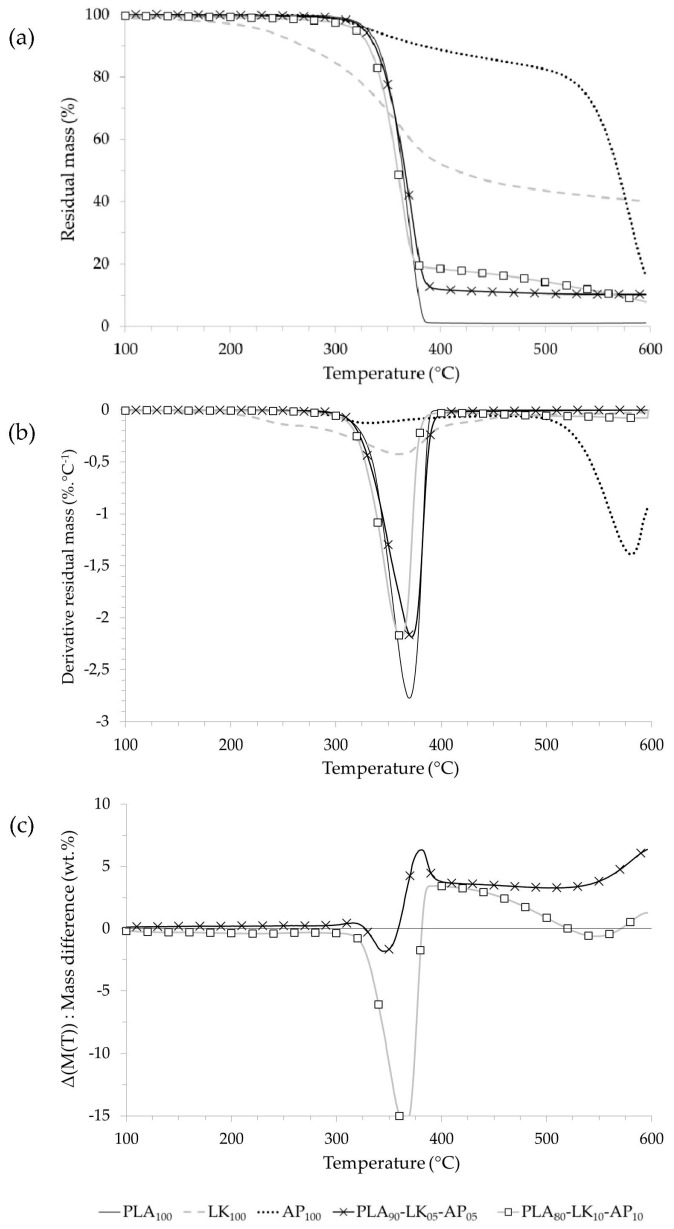
(**a**) TG; (**b**) DTG; and (**c**) mass difference curves of PLA-LK-AP composites.

The thermal behavior as well as the determination of the main degradation peaks of PLA, LK, and AP and composites are shown in Figure 3, Figure 4 and Figure 5, respectively, and Table 5.

On the one hand, lignin decomposition starts from 230 °C with a low rate (~0.4% °C^−1^ at 360 °C) and results in a high quantity of residue (~40% at 600 °C) due to the formation of polycyclic aromatic hydrocarbons as determined by Sharma et al. [39]. On the other hand, PLA degradation completely decomposes with no residue in one quick step which starts at 309 °C (5% weight loss) to finish up at 390 °C [40,41]. The PLA-LK blends show only one degradation step. The temperature at 5% weight loss of the composite samples is slightly lower than that of neat PLA, and in particular for the sample labeled PLA_80_-LK_20_, due to the LK decomposing earlier than the PLA. The destabilization of PLA in the presence of LK for the samples loaded at 5 and 10 wt % can be observed on the mass difference curves. Between 350 and 400 °C, the experimental residual masses of samples labeled PLA_95_-LK_05_ and PLA_90_-LK_10_ reach their lowest values (−10%). Above 400 °C, PLA-LK composites present a residue due to the charring capacity of LK. This residue is proportionately related to the loading content.

This residue at 500 °C is proportional to the initial loading of LK and corresponds to the LK residue, because the mass difference curve of composites show no positive interaction above 500 °C.

The thermal decomposition of ammonium polyphosphate occurs in two consecutive steps (Figure 4) [42]. The first degradation step from 330 °C corresponds to the release of ammonia and phosphoric acid which lead to polyphosphoric acid formation. From 500 °C, the second degradation step is related to the dehydratation of polyphosphoric acid to form phosphorus oxides. The profiles of thermal degradation of PLA blends are weakly influenced by the addition of AP (at 5 wt % and 10 wt %), which is illustrated by the fact that the curves are close to that of the neat PLA. The amount of residue at 500 °C is relatively low, 5% for 10 wt % of AP.

The mass difference curves for PLA_95_-AP_05_ and PLA_90_-AP_10_ show slight thermal stabilization between 300 °C and 400 °C followed by a thermal destabilization. Nevertheless, these mass differences are between +5% and −5%, and thus it can be concluded that interactions between PLA and AP that occur in the degradation of blends are weak. The thermal degradation of PLA-LK-AP composites is presented in Figure 5. All of the blends (PLA_90_-LK_05_-AP_05_ and PLA_80_-LK_10_-AP_10_) exhibit single-stage decomposition, as does the neat PLA. The degradation of PLA-LK-AP composites starts at a lower temperature than PLA and PLA-LK blends. This difference depends on the AP content, thus for the 10 wt %, PLA_80_-LK_10_-AP_10_ sample, the onset temperature shifts to 10 °C lower than neat PLA (~10 °C). The mass difference curve of the PLA_80_-LK_10_-AP_10_ sample (Figure 5) underlines the fact that the LK/AP combination destabilizes the blend before the main degradation of the PLA (Δ(*M*(*T*)) = −15% at 360 °C). However, the maximum decomposition rate of PLA_80_-LK_10_-AP_10_ (2.1% °C^−1^) at a temperature close to that of neat PLA is lower than that of neat PLA (2.8% °C^−1^). From 400 °C, this sample exhibits a thermally stable residue higher than 10 wt %. The mass difference curve at 400 °C indicates the positive interaction between the components of PLA-LK-AP composites (+5%). The sequence (destabilization–stabilization) is a standard thermal behavior of dehydration/charring systems formulated from polyhydric compounds and acid sources. It has already been showed that the presence of phosphate enhances the dehydration reactions of lignin with phosphate derivatives, leading to high amounts of char with condensed structures, which are not further degraded in low volatile compounds [43,44].

The UL-94 results are presented in Table 6. As expected, PLA specimens exhibit bad fire properties, and dripping occurs instantaneously, with the inflamed drops burning the cotton. An average sample mass of 39% is consumed. The PLA-LK blend has the same fire reaction behavior as neat PLA. The introduction of ammonium polyphosphate allows an improvement in the fire reaction of PLA blends. Furthermore, 10 wt % of AP is sufficient to avoid combustion, and therefore a lower weight loss has been observed, even if no charring effect was detected. The PLA-LK-AP blends have similar fire reaction behavior to that of PLA-AP composites ones. The presence of AP in the blends allows the creation of a char on the surface sample, and then a reduction in the weight loss.

**Figure 6:**
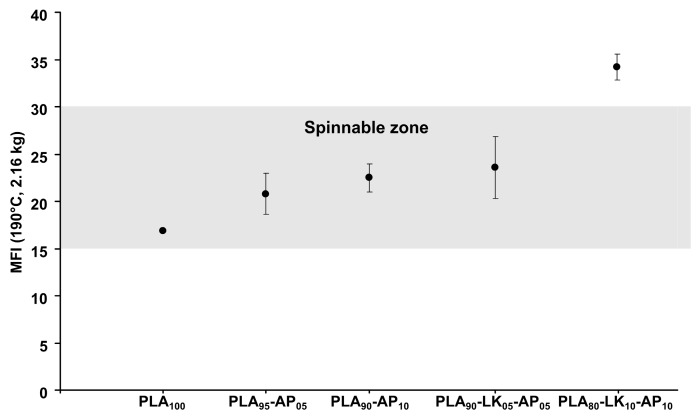
Melt flow index (190 °C, 2.16 kg) of PLA, PLA-AP and PLA-LK-AP pellets.

Figure 6 illustrates the influence of AP (at 5 and 10 wt %) on MFI values of PLA with and without LK, in order to determine the possibility of melt spinning. The incorporation of AP increases the MFI value, whereas the carbon source (LK) induces a fluidification of blends. This behavior may be due to the degradation of PLA macromolecular chains during the process. Even if AP does not act as a plasticizer since no modification of Tg_PLA_ has been observed (the data do not show any), the release of ammonia and phosphoric acid [42] above 200 °C leads to the degradation of PLA. The introduction of AP modifies the interactions PLA-LK in PLA-AP and AP-LK, and this result in the increase in fluidity. Any blend beyond the PLA_80_-LK_10_-AP_10_ sample is not spinnable for the cone calorimeter characterization.

**Figure 7:**
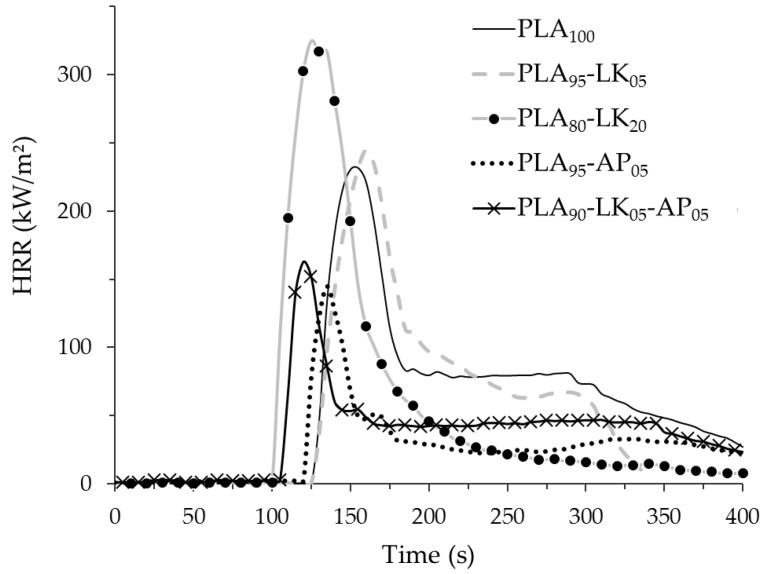
Heat release rate curves of the knitted fabrics (25 kW m^−2^).

Table 7 summarizes data obtained through cone calorimetry characterization of PLA, PLA_95_-AP_05_, PLA_95_-LK_05_, PLA_80_-LK_20_ and PLA_90_-LK_05_-AP_05_ knitted fabrics, and their HRR curves are plotted in Figure 7. The addition of lignin in PLA does not improve the composite fire reaction in comparison to neat PLA. For a 5 wt % LK loading content, the main flame reaction data parameters (t_ign_, PHRR, THR and MAHRE) are close to that of neat PLA and the difference for these parameters between PLA and PLA_95_-LK_05_ is included in the standard deviation of the average. Nevertheless, the addition of 5 wt % LK in PLA has significantly changed two fire parameters, the quantity of released smoke and the final residual mass. During the test, the visible charring effect due to the presence of lignin leads to the formation of a noticeable residue (13%) at the end of the cone experiment, which allows a decrease in the smoke quantity (−97%) in comparison to the neat PLA sample. For a 20 wt % LK loading content, the fire reaction is globally worse than one of the composite with 5 wt % LK loading content; especially the peak of heat release rate is 38% higher and the total smoke volume is six time higher in comparison with PLA_95_-LK_05_. The other fire reaction data (PHRR, THR and MAHRE) for PLA_80_-LK_20_ remain close to those for PLA_95_-LK_05_. The charring effect is also observed during the test for PLA_80_-LK_20_. However, increasing the amount of lignin in the PLA from 5% to 20% causes no increase in the final percentage of residual mass of the composite. Despite its charring behavior, lignin is a highly combustible material with a lower degradation temperature than neat PLA. Consequently, PLA with high lignin loading content as 20% presents worse fire reaction than neat PLA. Moreover, physical aspect of fiber—the formation of bumps onto the fiber surface due to the increase of LK content—should accentuate the bad fire properties. The introduction of 5 wt % of AP is sufficient to improve the fire reaction of PLA: −38% for PHRR and −92% for TSV. Nevertheless, a decrease of t_ign_ (−36 s) can be observed with 5 wt % of AP compare to neat PLA. This decrease almost remains in range of standard deviations of t_ign_ values. Even if the TGA characterizations under nitrogen on the PLA-AP composites point out neither the formation of a high quantity of char at high temperature nor positive interaction between PLA and AP, the residue of PLA95-AP05 combustion is important (27%).

The PLA-LK-AP blend exhibits the best fire reaction, but even if the PHRR is slightly higher in comparison to PLA-AP composite, the other fire parameters in the composite are better. In particular, the t_ign_ is closer to that of the neat PLA, and overall THR and MAHRE values are 56% and 43% lower than the reference. Besides, the combination of LK/AP allows an increase in the charring effect, which leads to the highest residue obtained at the end of the combustion.

## 4. Conclusions

In this work, the spinnability, thermal behavior as well as fire retardancy properties of PLA composites containing lignin and/or ammonium polyphosphate were studied by analyzing the variation of MFI values, thermal stability by TG, UL-94 and cone calorimeter experiments. The experimental results show that the incorporation of lignin decreased the MFI value, whereas with ammonium polyphosphate it was found to increase. These behaviors were ascribed to the creation of free volume in the PLA matrix, and the agglomeration of lignin particles. Furthermore, ammonium polyphosphate in the blends may not only degrade the macromolecular PLA chains, but also modify the interactions between all the compounds. From the results, only five blends have been spinnable to obtain multifilaments, i.e., PLA with 5 wt % of AP; PLA with 5, 10 and 20 wt % of LK; and the ternary blend PLA_90_-LK_05_-AP_05_. The thermal stability of the composites is slightly enhanced with the addition of ammonium polyphosphate, and the residue at 500 °C increases with lignin due to the charring capacity of this compound. Compared with PLA, PLA-AP composites show relatively low flammability according to the UL-94 test. The flame retardant properties of PLA knitted composite structures (PLA_95_-AP_05_, PLA_95_-LK_05_, PLA_80_-LK_20_, and PLA_90_-LK_05_-AP_05_) were investigated by cone calorimeter, showing that PLA-LK-AP cannot delay the ignition time, but does cause a significant reduction in the heat release rate due to the formation of char. Therefore, 5 wt % of LK and 5 wt % of AP are sufficient to obtain an efficient FR effect in PLA fabric.

## Figures and Tables

**Table 1:** Composition of the blends and associated labels.

Sample label	PLA (wt %)	Lignin Kraft (wt %)	Ammonium Polyphosphate (wt %)
PLA_100_	100	-	-
PLA_95_-LK_05_	95	5	-
PLA_90_-LK_10_	90	10	-
PLA_80_-LK_20_	80	20	-
PLA_95_-AP_05_	95	-	5
PLA_90_-AP_10_	90	-	10
PLA_90_-LK_05_-AP_05_	90	5	5
PLA_80_-LK_10_-AP_10_	80	10	10

**Table 2:** Spinning parameters of blends.

Blends	Extruder Temperature Profile (°C)							Roll 1		Roll 2		Draw Ratio
	T1	T2	T3	T4	T5	T6	T7	T (°C)	Speed (m min^−1^)	T (°C)	Speed (m min^−1^)	
PLA_100_	195	205	200	200	185	175	180	80	80	110	130	1.6
PLA_95_-LK_05_												
PLA_90_-LK_10_												
PLA_80_-LK_20_												
PLA_95_-AP_05_	195	205	200	200	185	180	180					
PLA_90_-LK_05_-AP_05_												

**Table 3:** Diameters and mechanical properties (tensile test) of monofilaments extracted from multifilaments.

	Diameter (μm)	Strain at Break (MPa)	Elongation at Break (%)	Tenacity (cN Tex^−1^)
PLA_100_	113 ± 2	277 ± 42	120 ± 47	21.9 ± 3.3
PLA_95_-LK_05_	141 ± 9	131 ± 20	224 ± 26	8.21 ± 1.2
PLA_90_-LK_10_	122 ± 18	70 ± 7	158 ± 43	5.4 ± 0.5
PLA_80_-LK_20_	152 ± 17	70 ± 11	75 ± 32	5.5 ± 0.8

**Table 4:** Thermal properties of PLA and PLA-LK samples.

Samples	Tg (°C)	T_CC_ Onset (°C)	T_CC_ Max (°C)	ΔH_CC_ (J g^−1^)	T_m_ Onset (°C)	T_m_ Max 1st Peak (°C)	T_m_ Max 2nd Peak (°C)	ΔH_m_ (J g^−1^)	χ_c_ (%)
PLA_100_ multifilaments	61.7	108.5	115.1	33.9	156.1	161.1	166.9	38.9	5.3
PLA_95_-LK_05_ extrudated	61.6	109.8	120.2	36.2	155.3	161.0	166.4	41.0	7.9
PLA_95_-LK_05_ multifilaments	60.4	104.1	112.2	37.7	155.0	160.9	166.4	40.0	2.6
PLA_90_-LK_10_ extrudated	61.5	110.6	122.9	38.1	155.1	161.8	166.1	38.5	0.4
PLA_90_-LK_10_ multifilaments	59.7	106.5	125.1	33.3	153.4	159.7	165.1	34.1	0.9
PLA_80_-LK_20_ extrudated	62.2	119.3	137.4	10.3	157.5	163.5	-	10.5	0.3
PLA_80_-LK_20_ multifilaments	55.5	111.0	127.0	32.8	151.1	158.0	163.7	36.0	4.3

**Table 5:** Thermogravimetry data of PLA, LK, AP, PLA-LK, PLA-AP and PLA-LK-AP samples.

Samples	T_onset5%_ (°C)	T_max_ (°C)	Residue at 500 °C (%)
PLA_100_	330.7	370.5	1.1
LK_100_	232.1	357.8	43.5
AP_100_	334.6	580.7	82.4
PLA_95_-LK_05_	329.1	366.1	4.2
PLA_90_-LK_10_	329.0	363.7	6.4
PLA_80_-LK_20_	314.5	369.6	9.8
PLA_95_-AP_05_	333.6	372.7	2.6
PLA_90_-AP_10_	333.0	373.0	5.0
PLA_90_-LK_05_-AP_05_	327.7	372.3	10.5
PLA_80_-LK_10_-AP_10_	320.0	362.0	14.3

**Table 6:** Results of UL-94 vertical test.

Samples	Average Combustion Time (s)	Mass Lost (%)	Ignition of the Cotton	Classification
PLA_100_	17 ± 5	39.1 ± 15.0	Yes	V2
PLA_95_-AP_05_	1 ± 1	6.7 ± 2.1	No	V0
PLA_90_-AP_10_	0	1.4 ± 0.6	No	V0
PLA_90_-LK_10_	20 ± 2	34.1 ± 4.7	Yes	V2
PLA_90_-LK_05_-AP_05_	0	4.5 ± 2.3	No	V0
PLA_80_-LK_10_-AP_10_	0	6.3 ± 2.0	No	V0

**Table 7:** Results of cone calorimeter test for the knitted fabrics (25 kW m^−2^).

Samples	t_ign_ (s)	PHRR (kW m^−2^)	THR (MJ m^−2^)	TSV (m^2^)	MAHRE (kW m^−2^)	Residue (%)
PLA_100_	126 ± 5	231.5 ± 4	25 ± 2	175 ± 15	62 ± 6	4 ±2
PLA_95_-AP_05_	90 ± 21	143 ± 2	18 ± 7	13 ± 7	42 ± 11	27 ± 4
PLA_95_-LK_05_	143 ± 11	228 ± 24	17 ± 2	5 ± 1	57 ± 4	13± 2
PLA_80_-LK_20_	119 ± 15	315 ± 14	24 ± 3	32 ± 9	78 ± 6	14 ± 2
PLA_90_-LK_05_-AP_05_	112 ± 6	157 ± 8	11 ± 1	9 ± 1	35 ± 3	36 ± 3
